# Supplementary material for: Echinococcus granulosus-Induced Liver Damage Through Ferroptosis in Rat Model
Source: Cells. 2025 Feb 22;14(5):328. doi: 10.3390/cells14050328 (PMC11898441; doi:10.3390/cells14050328)
Supplement: Supplementary file 1 [file cells-14-00328-s001.zip › Figures S1-S5 legend .pdf]

**Fig.S1.** PCR amplification of mitochondrial DNA *COX1* gene in *Echinococcus granulosus* （M：DNA Marker2000bp；1、2：*COX1* gene）
Collect PSCs from the hepatic cysts of sheep with *Echinococcus granulosus*, extract them, and amplify the PSC parasite DNA by PCR gene fragment amplification using the mitochondrial *COX1* gene. Obtain a 936 bp fragment that matches the expected fragment size, and perform gene sequencing on the PCR product.

**Fig. S2.** Pathological characteristics of rat liver cyst cystic fibrosis at different stages of infection by PSCs (germinal layer, keratinization layer, inflammatory cell zone are indicated by Black, Red, white and blue arrows , n=6 Rat/group , the scale bars represent 100 μm and 50 μm).
Masson Cytochemical staining observation showed that the smooth muscle of the portal vein and central vein in normal rat liver tissue was stained blue (Fig 3-A, B); One month old rats were infected with PSCs, which caused extensive damage to liver cells and stimulated the expression and differentiation of fibrin in peripheral liver tissue, extending the lesion to wrap around the interstitial space of PSCs (Fig 3-C, D); Three month old rats infected with PSCs showed lighter staining of liver cells around the cyst, disappearance of cell nuclei, significant proliferation of fibrous tissue in the cyst wall, and extended growth of damaged liver cells to the periphery, forming a more obvious layered structure (Fig 3-E, F). In rats infected with the original head larvae for 6 months, the liver cysts developed and matured, with increased deposition and darkening of fibrin on the cyst wall. The fibrin on the cyst wall extended to the peripheral liver tissue and gradually replaced the damaged liver cells (Fig 3-G, H).

**Fig. S3.** Statistics and analysis of the number of inflammatory cells in hepatic cysts. (germinal layer, keratinization layer, inflammatory cell zone are indicated by Black, Red, white and blue arrows , n=6 Rat/group , the scale bars represent 100 μm and 20 μm).

**Fig S4:** KEGG Pathways P3VP1.

**Fig.S5.** Pathological characteristics of rat liver cyst cystic fibrosis after Ferrostatin-1 intervention in vivo (PSCs，keratinization layer, inflammatory cell zone are indicated by Black, white and blue arrows , n=6 Rat/group , the scale bars represent 100 μm and 50 μm).
Masson cell chemical staining observation showed that after Ferrostatin-1 intervention in vivo, the fibrous connective tissue within the lesion became loose and the staining became lighter, with a clear boundary with peripheral liver cells.
